# Exposure misclassification bias in the estimation of vaccine effectiveness

**DOI:** 10.1371/journal.pone.0251622

**Published:** 2021-05-13

**Authors:** Ulrike Baum, Sangita Kulathinal, Kari Auranen

**Affiliations:** 1 Department of Health Security, Finnish Institute for Health and Welfare, Helsinki, Finland; 2 Department of Mathematics and Statistics, University of Helsinki, Helsinki, Finland; 3 Department of Mathematics and Statistics, University of Turku, Turku, Finland; 4 Department of Clinical Medicine, University of Turku, Turku, Finland; Universitat Politecnica de Catalunya, SPAIN

## Abstract

In epidemiology, a typical measure of interest is the risk of disease conditional upon exposure. A common source of bias in the estimation of risks and risk ratios is misclassification. Exposure misclassification affects the measurement of exposure, i.e. the variable one conditions on. This article explains how to assess biases under non-differential exposure misclassification when estimating vaccine effectiveness, i.e. the vaccine-induced relative reduction in the risk of disease. The problem can be described in terms of three binary variables: the unobserved true exposure status, the observed but potentially misclassified exposure status, and the observed true disease status. The bias due to exposure misclassification is quantified by the difference between the naïve estimand defined as one minus the risk ratio comparing individuals observed as vaccinated with individuals observed as unvaccinated, and the vaccine effectiveness defined as one minus the risk ratio comparing truly vaccinated with truly unvaccinated. The magnitude of the bias depends on five factors: the risks of disease in the truly vaccinated and the truly unvaccinated, the sensitivity and specificity of exposure measurement, and vaccination coverage. Non-differential exposure misclassification bias is always negative. In practice, if the sensitivity and specificity are known or estimable from external sources, the true risks and the vaccination coverage can be estimated from the observed data and, thus, the estimation of vaccine effectiveness based on the observed risks can be corrected for exposure misclassification. When analysing risks under misclassification, careful consideration of conditional probabilities is crucial.

## Introduction

Measurement error and misclassification result into information bias, i.e. a systematic error in the estimator of an exposure’s effect on an outcome [1]. If the probability of misclassification in one variable does not depend on the level of other variables, misclassification is said to be non-differential. When estimating vaccine effectiveness, the exposure and outcome of interest are vaccination and occurrence of a given vaccine-preventable disease, respectively. The aim of measuring vaccine effectiveness is to quantify the relative reduction in the disease’s risk or rate attributable to vaccination [2, 3].

Under the assumption that the disease is rare or that the vaccine offers complete protection to a subset of the vaccinated individuals while leaving the rest unaffected, vaccine effectiveness can be estimated as one minus the risk ratio [2]. The two (cumulative) risks that are compared by the risk ratio are the two conditional probabilities of the disease in those who have been vaccinated and those who have not. While outcome misclassification affects the measurement of outcome conditional on exposure, exposure misclassification affects the measurement of exposure, i.e. the variable one should condition on.

De Smedt et al. [4] studied non-differential outcome and exposure misclassification bias in the estimation of vaccine effectiveness based on the risk ratio. Regarding outcome misclassification, their analysis is complete and in line with previous literature [5, 6]. Their mathematical presentation of the bias caused by exposure misclassification is, however, not complete. We here provide a detailed derivation of the bias, emphasising the need for proper conditioning on a potentially misclassified exposure status. Finally, we show how the estimation of vaccine effectiveness can be adjusted for exposure misclassification if the sensitivity and specificity of exposure measurement are known or estimable from external sources. We use the register-based estimation of influenza vaccine effectiveness in the Finnish elderly as an example [7].

## Methods

A person is considered either vaccinated if a vaccine under study has been administered or unvaccinated if the vaccine has not been administered. We refer to this classification as the true vaccination status. In a study, however, the collected vaccination information might be subject to underreporting, omission, coding error or inaccurate recall, so a person’s true vaccination status is unknown to the investigator. We therefore refer to the classification of individuals into vaccinated and unvaccinated based on study data as the observed but potentially misclassified vaccination status.

**Fig 1:**
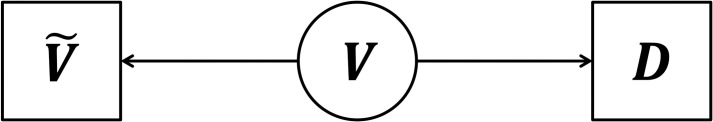
Exposure misclassification model. *D*: observed true disease status, binary; *V*: unobserved true vaccination status, binary; $\tilde{V}$: observed and potentially misclassified vaccination status, binary.

The problem of studying vaccine effectiveness under exposure misclassification can be described in terms of three binary variables: the unobserved true vaccination (exposure) status (*V*), the observed but potentially misclassified vaccination (exposure) status ($\tilde{V}$), and the observed true disease (outcome) status (*D*). Fig 1 shows how these three variables are related.

The association of interest is *V*→*D*, i.e. the risk (probability) of disease conditional on the true vaccination status *P*(*D* = 1|*V*). However, from observed data one can infer directly only the association $\tilde{V}\to D$, i.e. the risk of disease conditional on the observed vaccination status $P(D=1|\tilde{V})$.

Assuming the absence of other sources of bias, it follows from the dependencies depicted in Fig 1 that the joint probability of the three model quantities is

$$P(V,\tilde{V},D)=P(V)∙P(\tilde{V}|V)∙P(D|V,\tilde{V})=P(V)∙P(\tilde{V}|V)∙P(D|V).$$ (1)

Let *γ* = *P*(*V* = 1) be the true vaccination coverage, i.e. the proportion of truly vaccinated individuals (Table 1). Exposure misclassification is quantified by the sensitivity and specificity of exposure measurement. The sensitivity is the probability $SE=P(\tilde{V}=1|V=1)$ of measuring correctly the vaccination status of a vaccinated individual. Correspondingly, the specificity is the probability $SP=P(\tilde{V}=0|V=0)$ of measuring correctly the vaccination status of an unvaccinated individual. The risks of disease in the truly vaccinated and the truly unvaccinated are *π*_1_ = *P*(*D* = 1|*V* = 1) and *π*_0_ = *P*(*D* = 1|*V* = 0), respectively. The effect measure of interest is the vaccine effectiveness defined by the estimand *VE* = 1−*π*_1_/*π*_0_ (Table 1).

**Table 1:** Notation.

Parameter	Symbol	Explanation
*P*(*V* = 1)	*γ*	True vaccination coverage
$P(\tilde{V}=1|V=1)$	*SE*	Sensitivity
$P(\tilde{V}=0|V=0)$	*SP*	Specificity
*P*(*D* = 1|*V* = 1)	*π*_1_	Risk in the truly vaccinated
*P*(*D* = 1|*V* = 0)	*π*_0_	Risk in the truly unvaccinated
1−*π*_1_/*π*_0_	*VE*	Vaccine effectiveness
$P(\tilde{V}=1)$		Observed vaccination coverage
$P(D=1|\tilde{V}=1)$	*p*_1_	Risk in individuals observed as vaccinated
$P(D=1|\tilde{V}=0)$	*p*_0_	Risk in individuals observed as unvaccinated
1−*p*_1_/*p*_0_		Naïve estimand of vaccine effectiveness
(1−*p*_1_/*p*_0_)− *VE*	Δ	Bias

*D*: observed true disease status, binary; *V*: unobserved true vaccination status, binary; $\tilde{V}$: observed and potentially misclassified vaccination status, binary.

Since the true vaccination status and, therefore, *γ*, *π*_1_ and *π*_0_ are unobserved, we can only measure the observed vaccination coverage, $P(\tilde{V}=1)$, and the risks of disease conditionally upon the observed but potentially misclassified vaccination status, $p_{1}=P(D=1|\tilde{V}=1)$ and $p_{0}=P(D=1|\tilde{V}=0)$ (Table 1).

It follows from the conditional independence of *D* and $\tilde{V}$ given *V*, as expressed in Eq (1), that

$$\begin{array}{l} p_{1}=P(D=1|\tilde{V}=1)\\ \;=P(D=1,V=1|\tilde{V}=1)+P\left(D=1,V=0|\tilde{V}=1\right)\\ \;=P(V=1|\tilde{V}=1)\cdot \pi_{1}+P(V=0|\tilde{V}=1)\cdot \pi_{0} \end{array}$$ (2A)

and

$$p_{0}=P(V=1|\tilde{V}=0)\cdot \pi_{1}+P(V=0|\tilde{V}=0)\cdot \pi_{0}.$$ (3A)

The observed risks *p*_1_ and *p*_0_ can thus be interpreted as weighted averages of the true risks *π*_1_ and *π*_0_. The four weights, each giving the probability of the true vaccination status conditioned upon the observed vaccination status, can be expressed in terms of three parameters, *γ*, *SE* and *SP*:

$$\begin{array}{l} p_{1}=P(V=1|\tilde{V}=1)\cdot \pi_{1}+P(V=0|\tilde{V}=1)\cdot \pi_{0}\\ \;=\frac{P(V=1,\tilde{V}=1)}{P(\tilde{V}=1)}\cdot \pi_{1}+\frac{P(V=0,\tilde{V}=1)}{P(\tilde{V}=1)}\cdot \pi_{0}\\ \;=\frac{P(\tilde{V}=1|V=1)\cdot P(V=1)\cdot \pi_{1}+P(\tilde{V}=1|V=0)\cdot P(V=0)\cdot \pi_{0}}{P(\tilde{V}=1)}\\ \;=\frac{SE\cdot \gamma \cdot \pi_{1}+(1-SP)\cdot (1-\gamma )\cdot \pi_{0}}{SE\cdot \gamma +(1-SP)\cdot (1-\gamma )} \end{array}$$ (2B)

and

$$p_{0}=\frac{(1-SE)\cdot \gamma \cdot \pi_{1}+SP\cdot (1-\gamma )\cdot \pi_{0}}{1-SE\cdot \gamma -(1-SP)\cdot (1-\gamma )}.$$ (3B)

If both the sensitivity *SE*≈0.5 and specificity *SP*≈0.5, i.e. if the observed vaccination status $\tilde{V}=1$ is equally likely in truly vaccinated and unvaccinated individuals, the observed risks *p*_1_ and *p*_0_ are approximately identical (*p*_1_≈*p*_0_) and the vaccine effectiveness is not identifiable from the data. We therefore assume hereafter that *SE* and *SP* are away from 0.5.

Solving Eqs (2B) and (3B) for *π*_1_ and *π*_0_, we obtain

$$\pi_{1}=\frac{p_{1}∙SP∙\left(SE∙\gamma +\left(1-SP\right)∙\left(1-\gamma \right)\right)-p_{0}∙\left(1-SP\right)∙\left(1-SE∙\gamma -\left(1-SP\right)∙\left(1-\gamma \right)\right)}{\gamma ∙\left(SE+SP-1\right)}\mathrm{and}$$ (4)

$$\pi_{0}=\frac{p_{0}∙SE∙(1-SE∙\gamma -(1-SP)∙(1-\gamma ))-p_{1}∙(1-SE)∙(SE∙\gamma +(1-SP)∙(1-\gamma ))}{\left(1-\gamma )∙(SE+SP-1\right)}.$$ (5)

The estimand *VE* follows as

$$VE=1-\frac{\pi_{1}}{\pi_{0}}=1-\frac{1-\gamma }{\gamma }∙\frac{p_{1}∙SP∙(SE∙\gamma +(1-SP)∙(1-\gamma ))-p_{0}∙(1-SP)∙(1-SE∙\gamma -(1-SP)∙(1-\gamma ))}{p_{0}∙SE∙(1-SE∙\gamma -(1-SP)∙(1-\gamma ))-p_{1}∙(1-SE)∙(SE∙\gamma +(1-SP)∙(1-\gamma ))}.$$ (6)

This expression still involves the unobserved true vaccination coverage *γ*. However, by solving $P(\tilde{V}=1)=SE∙\gamma +(1-SP)∙(1-\gamma )$ for *γ*, *γ* can be expressed in terms of the observed vaccination coverage $P(\tilde{V}=1)$, sensitivity *SE* and specificity *SP*:

$$\gamma =P(V=1)=\frac{P(\tilde{V}=1)+SP-1}{SE+SP-1}.$$ (7)

Since *VE* thus depends only on parameters that can be estimated from the observed data ($P(\tilde{V}=1),\;p_{1},\;p_{0}$) or might be known or estimable from external sources (*SE*, *SP*), the vaccine effectiveness can be estimated even under exposure misclassification. In the absence of exposure misclassification (*SE* = *SP* = 1), Eq (6) simplifies to the standard expression of vaccine effectiveness based on observed risks. Under exposure misclassification, however, this naïve estimand, (1−*p*_1_/*p*_0_), differs from the correct estimand, *VE*. The difference Δ of the two estimands quantifies the bias due exposure misclassification:

$$\begin{array}{l} \Delta =(1-\frac{p_{1}}{p_{0}})-VE=(1-\frac{p_{1}}{p_{0}})-(1-\frac{\pi_{1}}{\pi_{0}})\\ \;=\frac{\pi_{1}}{\pi_{0}}-\frac{SE∙\gamma ∙\pi_{1}+(1-SP)∙(1-\gamma )∙\pi_{0}}{(1-SE)∙\gamma ∙\pi_{1}+SP∙(1-\gamma )∙\pi_{0}}∙\underset{X}{\underset{︸}{\frac{1-SE∙\gamma -(1-SP)∙(1-\gamma )}{SE∙\gamma +(1-SP)∙(1-\gamma )}}}. \end{array}$$ (8)

The last term, *X*, is due to the denominators in Eqs (2B) and (3B) and results from correct conditioning on the observed vaccination status. Of note, De Smedt et al. [4] expressed the observed risks *p*_1_ and *p*_0_ omitting these denominators and their mathematical presentation of bias Δ thus misses term *X*.

We here graphically present the difference between the two estimands, (1−*p*_1_/*p*_0_) and *VE*, for different scenarios using Eqs (2B), (3B) and (6). We set *π*_1_ = 0.045 and *π*_0_ = 0.15, implying that the true value of the vaccine effectiveness equals 0.7. The values for the true vaccination coverage, sensitivity and specificity are taken from *γ*∈(0,1), *SE*∈{0.6,0.8,1}, and *SP*∈{0.6,0.8,1}.

In addition, we demonstrate how the estimation of vaccine effectiveness can be adjusted for exposure misclassification taking the register-based estimation of influenza vaccine effectiveness in the Finnish elderly (aged 65 years and above) in 2016/17 as an example [7]. All individuals in the study population are classified as vaccinated or unvaccinated based on their records in the Finnish vaccination register giving the observed and potentially misclassified vaccination status $\tilde{V}$. Assuming the register’s specificity to be perfect, its sensitivity *SE* is first evaluated as the ratio of the observed vaccination coverage in the subpopulation of individuals aged 70 to 79 years and the corresponding vaccination coverage in a representative survey [8], which we assume to reflect the true vaccination coverage in that subpopulation. We then use Eq (7) to calculate the true vaccination coverage *γ* for the whole study population and Eq (6) to assess the vaccine effectiveness using the values of *γ*, *SE* and *SP* = 1, as well as the observed influenza risks *p*_1_ and *p*_0_.

## Results

Five parameters determine the magnitude of exposure misclassification bias: the risks of the disease in the truly vaccinated (*π*_1_) and the truly unvaccinated (*π*_0_), the sensitivity (*SE*), the specificity (*SP*) and the true vaccination coverage (*γ*). The last term, *X*, in Eq (8) arising from proper conditioning depends on three parameters: true vaccination coverage, sensitivity and specificity. Because *X* describes a ratio of two probabilities, its magnitude ranges from 0 to infinity.

**Fig 2:**
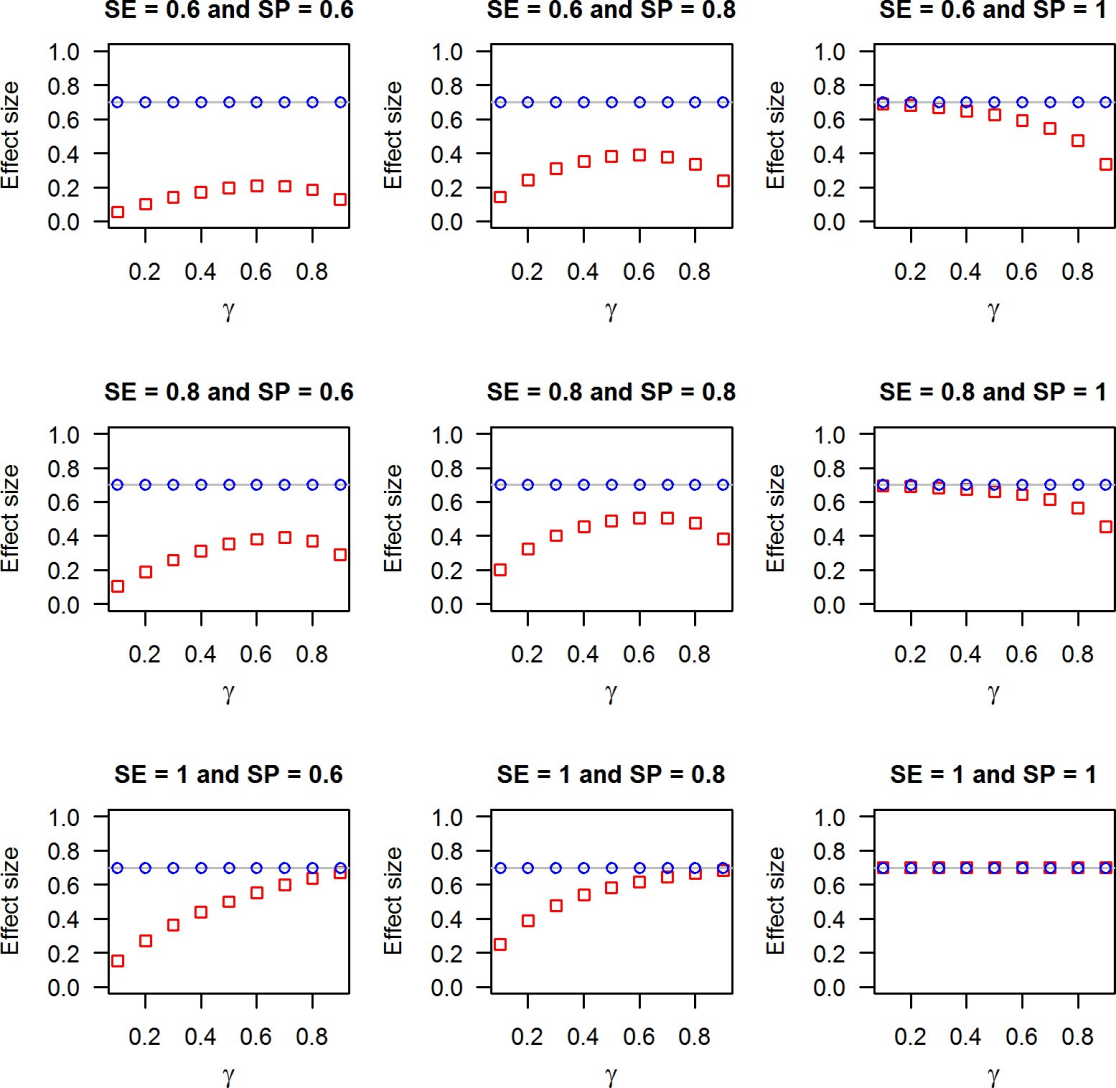
**Effect size measured by the estimands (1−*p***_**1**_**/*p***_**0**_**) (red squares) and *VE* (blue circles) under non-differential exposure misclassification.** Data points were calculated using Eqs (2B), (3B) and (6), *π*_1_ = 0.045, *π*_0_ = 0.15 and *γ*, *SE* and *SP* as given in the panels. The horizontal grey line marks the true vaccine effectiveness.

Fig 2 presents the effect size measured by the estimands (1−*p*_1_/*p*_0_) and *VE* under non-differential exposure misclassification. While *VE* accurately measures the vaccine effectiveness at 0.7, (1−*p*_1_/*p*_0_) does not quantify the vaccine effectiveness correctly unless both sensitivity and specificity are perfect. The vertical distance between the two effect measures depicted in Fig 2 marks the magnitude of the bias. The bias is always negative. As imperfect sensitivity leads to misclassification of the truly vaccinated, its impact is strongest when the true vaccination coverage is high. Vice versa, the impact of imperfect specificity, which leads to misclassification of the truly unvaccinated, is strongest when the true vaccination coverage is low.

In the example from the 2016/17 influenza season, the vaccination coverage in elderly aged 70 to 79 years was 51% according to the register [9] and 64% according to the survey [8]. The sensitivity (*SE*) of the register-based exposure measurement was thus estimated at 80% (= 51%/64%∙100%). The observed vaccination coverage in the study population, $P(\tilde{V}=1)$, was 47% and the estimated cumulative risks (*p*_1_ and *p*_0_) were 16% and 20%, respectively [7]. It follows from Eqs (7) and (6) that the true vaccination coverage (*γ*) was 59% and the vaccine effectiveness (*VE*) was 24%. The vaccine effectiveness was thus 4 percentage points higher than the naïve estimate, (1−*p*_1_/*p*_0_), would have suggested.

## Discussion

In this article, we derived an expression for the magnitude of non-differential exposure misclassification bias in the estimation of vaccine effectiveness based on risk ratios. The bias depends on five factors: true vaccination coverage, sensitivity and specificity of exposure measurement, and the risk of the disease of interest in the truly vaccinated and the truly unvaccinated. If the sensitivity and specificity are known or estimable from external sources, Eq (6) can be used to correct the estimation of vaccine effectiveness based on the observed risks for exposure misclassification. In absence of exact information about the sensitivity and specificity of exposure measurement, Eq (8) can be used for assessing the potential magnitude of bias given a range of plausible values.

Our findings are in line with previous literature. In contrast to non-differential outcome misclassification, non-differential exposure misclassification leads to bias [4]. The result that this bias is always negative must not be misinterpreted as a generic rule that any naïve estimate derived under exposure misclassification would be an underestimate of vaccine effectiveness. Due to random error, a single naïve estimate can under- or overestimate the true effect [10, 11]. This random error remains even after adjusting for the bias as described in this paper. Which of the two errors dominates depends on the true values of the underlying parameters.

We described the problem of studying vaccine effectiveness under exposure misclassification and absence of other sources of bias in terms of three binary variables and formulated the joint probabilities of the three model quantities. Our exposure misclassification model (Fig 1) is a special case of the model presented by Tang et al. [12] for misclassification in both exposure and outcome. This approach of jointly modelling all observables and conditioning on the actually observed variables facilitates the connection between conditional probabilities and standard parameters such as sensitivity, specificity and risks, avoiding fallacies like the one pointed out in this paper. Moreover, if exposure misclassification should be differential, it can be easily incorporated in the initial model by allowing dependence of the sensitivity and specificity on the disease status or other variables.

This article pointed out the importance of proper conditioning. If misclassification in the exposure status is not accurately taken into account, expressions quantifying the magnitude of bias will be wrong. Based on the correct equations, a potentially biased vaccine effectiveness (or more generally any risk ratio) estimate can be adjusted for exposure misclassification. Nevertheless, this requires that the sensitivity and specificity of exposure measurement are known or are estimable from external sources.

## References

[1] Hill HA, Kleinbaum DG. Bias in Observational Studies. In: Armitage P, Colton T, editors. Encyclopedia of Biostatistics. 2nd ed. Hoboken: John Wiley and Sons, Ltd; 2005.

[2] Smith PG, Rodrigues LC, Fine PE. Assessment of the protective efficacy of vaccines against common diseases using case-control and cohort studies. Int J Epidemiol 1984 3 01;13(1):87–93. 10.1093/ije/13.1.87 6698708

[3] Hanquet G, Valenciano M, Simondon F, Moren A. Vaccine effects and impact of vaccination programmes in post-licensure studies. Vaccine 2013 11 19;31(48):5634–5642. 10.1016/j.vaccine.2013.07.006 23856332

[4] De Smedt T, Merrall E, Macina D, Perez-Vilar S, Andrews N, Bollaerts K. Bias due to differential and non-differential disease- and exposure misclassification in studies of vaccine effectiveness. PLoS One 2018 6 15;13(6):e0199180. 10.1371/journal.pone.0199180 29906276 PMC6003693

[5] Orenstein EW, De Serres G, Haber MJ, Shay DK, Bridges CB, Gargiullo P, et al. Methodologic issues regarding the use of three observational study designs to assess influenza vaccine effectiveness. Int J Epidemiol 2007 6 01;36(3):623–631. 10.1093/ije/dym021 17403908

[6] Jackson ML, Rothman KJ. Effects of imperfect test sensitivity and specificity on observational studies of influenza vaccine effectiveness. Vaccine 2015 3 10;33(11):1313–1316. 10.1016/j.vaccine.2015.01.069 25659280 PMC5934991

[7] Baum U, Kulathinal S, Auranen K. Mitigation of biases in estimating hazard ratios under non-sensitive and non-specific observation of outcomes-applications to influenza vaccine effectiveness. Emerg Themes Epidemiol 2021 1 14;18(1):1–z. 10.1186/s12982-020-00091-z 33446220 PMC7807790

[8] Koponen P, Borodulin K, Lundqvist A, Sääksjärvi K, Koskinen S, editors. Terveys, toimintakyky ja hyvinvointi Suomessa: FinTerveys 2017-tutkimus. Helsinki: Finnish Institute for Health and Welfare; 2018.

[9] Hergens MP, Baum U, Brytting M, Ikonen N, Haveri A, Wiman A, et al. Mid-season real-time estimates of seasonal influenza vaccine effectiveness in persons 65 years and older in register-based surveillance, Stockholm County, Sweden, and Finland, January 2017. Euro Surveill 2017 2 23;22(8):30469. 10.2807/1560-7917.ES.2017.22.8.30469 28251891 PMC5356437

[10] Jurek AM, Greenland S, Maldonado G, Church TR. Proper interpretation of non-differential misclassification effects: expectations vs observations. Int J Epidemiol 2005 6 01;34(3):680–687. 10.1093/ije/dyi060 15802377

[11] Whitcomb BW, Naimi AI. Things don’t always go as expected: the example of non-differential misclassification of exposure—bias and error. Am J Epidemiol 2020 2 21;189(5):365–368. 10.1093/aje/kwaa020 32080716

[12] Tang L, Lyles RH, King CC, Celentano DD, Lo Y. Binary regression with differentially misclassified response and exposure variables. Stat Med 2015 4 30;34(9):1605–1620. 10.1002/sim.6440 25652841 PMC4418038

